# Inflammasome signaling proteins as biomarkers of COVID-19

**DOI:** 10.3389/fimmu.2023.1014665

**Published:** 2023-04-24

**Authors:** Roey Hadad, Robert W. Keane, Juan Pablo de Rivero Vaccari

**Affiliations:** ^1^ Department of Physiology and Biophysics, University of Miami Miller School of Medicine, Miami, FL, United States; ^2^ Department of Neurological Surgery and The Miami Project to Cure Paralysis, University of Miami Miller School of Medicine, Miami, FL, United States

**Keywords:** inflammasome, biomarkers, COVID-19, caspase-1, ASC, IL-1

## Abstract

**Introduction:**

One of the main characteristics of COVID-19 is an exacerbated inflammatory response that results in cardiometabolic complications and dysfunction in the nervous system. Moreover, these complications may extend beyond the period of active SARS-CoV2 infection and even extend over a year. Thus, it is important to better understand the contribution of the inflammatory responses in COVID-19 patients, not just in the acute phase but also after the infection has subsided.

**Methods:**

We measured the protein levels of inflammasome signaling proteins using Simple Plex microfluidics technology in patients with an active SARS-CoV2 infection and in recovered patients to determine their potential use as biomarkers of COVID-19. We carried out statistical analyses to identify which proteins were increased in COVID-19 patients with active infection and in recovered patients. The receiver operating characteristics (ROC) were calculated for each analyte to determine their potential fit as biomarkers.

**Results:**

The inflammasome proteins caspase-1, apoptosis-associated speck-like protein containing a caspase recruitment domain (ASC), interleukin (IL)-1β and IL-18 were elevated in the plasma of patients with active infection and remained elevated after the infection was resolved for approximately 2 months after. Levels of caspase-1 and ASC continued to increase long after patients had recovered from the infection. Furthermore, when measuring biomarkers of inflammation during active infection, analyses with area under the curve (AUC) values above 0.75 indicated that caspase-1, ASC, IL-1β and IL-18 are reliable biomarkers of the inflammatory response during active COVID-19 infection. Moreover, when measuring biomarkers of inflammation after recovery from active infection, caspase-1 and ASC presented AUC values above 0.9.

**Discussion:**

These findings indicate that inflammasome signaling proteins can be used to reliably monitor the inflammatory innate immune response in COVID-19 patients.

## Introduction

Coronaviruses (CoVs) are responsible for diseases such as severe acute respiratory syndrome (SARS), Middle East respiratory syndrome (MERS), and most recently, coronavirus disease-2019 (COVID-19) (1). CoVs are enveloped positive-sense single stranded RNA viruses from the genus Betacoronavirus and the Coronaviridiae family (2). COVID-19 remains the most studied disease associated with CoVs infections. However, there is still a lack of knowledge as to how the inflammatory response contributes to COVID-19 pathology.

In this study, we examined the biomarker potential of proteins associated with a key feature of COVID-19, namely inflammation. The inflammatory response in COVID-19 is severely exacerbated acutely during infection. A key contributor to the inflammatory response after CoVs is the inflammasome, a regulator of the inflammatory cytokines interleukin (IL)-1β and IL-18. Several proteins involved in the life cycle of CoVs during infection such as Viroporin E, open reading frame3a (ORF3a) and ORF8b have been shown to play a role in the activation of the inflammasome (2); thereby, indicating the importance of the inflammasome in the innate immune response after SARS-Co-V2 infections.

In addition to its role in COVID-19 pathology, the inflammasome contributes to pathomechanisms of acute respiratory distress syndrome (ARDS); ventilator-induced lung injury (VILI); ventilator-associated pneumonia (VAP); systemic complications associated with cytokine release syndrome (CRS), such as cardiac pressure overload, cardiac arrhythmia, hypertension, inflammatory conditions in the gastrointestinal tract, pancreatic islet cell damage as well as cerebrovascular and renal complications, and disseminated intravascular coagulation (DIC) (2).

Inflammatory proteins have been previously analyzed in the blood of COVID-19 infected patients (3). Increased levels of IL-6 and C-reactive protein (CRP) have been found in blood samples from COVID-19 patients with peak levels measured at 3 days after hospitalization in critically ill survivors and a continuous increase in non-survivors (4). Moreover, levels of CRP, IL-6, IL-8, IL-10, tumor necrosis factor (TNF)-α and IL-2R showed a positive correlation with high-sensitivity cardiac troponin I, consistent with the contribution of the inflammatory response to cardiac injury present in COVID-19 patients (5). Another study identified higher protein levels of vascular endothelial growth factor (VEGF) at 24 hours after hospitalization (6).

We have previously shown that inflammasome proteins are reliable biomarkers of the inflammatory response in a variety of diseases including traumatic brain injury (7–10), multiple sclerosis (11), stroke (12), Alzheimer’s disease (13), non-alcoholic steatohepatitis (NASH) (14), age-related macular degeneration (15) and renal diseases (16). Given that a significant subset of COVID-19 patients experience long term sequelae from SARS-CoV2 infection (17, 18), it is important to understand not only the acute inflammatory response in COVID-19 patients, but also the chronic response that may contribute to a variety of systemic complications. Therefore, in this study, we investigated the biomarker potential of inflammasome signaling proteins in patients with active COVID-19 infections and patients who recovered from COVID-19 in order to determine the acute and more chronic inflammatory response in this patient population.

## Materials and methods

### Participants

Plasma samples from patients diagnosed with COVID-19, patients who recovered from COVID-19 and healthy controls never infected with COVID-19 were obtained from BioIVT (Hicksville, NY) (**Table 1**). Upon sample arrival in dry ice from BioIVT, samples were stored at -80oC until analyses. Control samples consisted of 47 patients (21 males (45%) and 26 females (55%)). Within this cohort of control subjects, 10 were Hispanic (21%), 18 were Black (38%) and 19 were Caucasian (41%). Age range was 24 to 61 years old, with a mean age of 38.40 years old and a median age of 35 years old. Samples from COVID-19 positive patients were collected between April 8th, 2020 and November 24th, 2020. Samples from patients who recovered from COVID-19 were collected between July 20th, 2020 and November 8th, 2020. Samples from healthy controls were obtained prior to 2019. COVID-19 samples were collected by SERATRIALS, LLC using IRB tracking number 20200828 approved on April 6th 2020. Subjects approved by informed consent for themselves to be enrolled in the study (no samples were collected if consent was needed from a legally authorized representative).

**Table 1:** Patients used in the study.

	COVID-19 Positive	COVID-19 Recovered
Sex		
Male	9 (31%)	10 (48%)
Female	20 (69%)	11 (52%)
Age		
Median	38	34
Range	19 to 67	18 to 58
Race		
White/Caucasian	25 (86%)	19 (90%)
Asian	2 (7%)	0
Black/African American	1 (3.5%)	1 (5%)
Other	1 (3.5%)	1 (5%)
Days from Diagnosis to collection		
Median	11	61.5
Range	1 to 24	28 to 217
Severity		
Asymptomatic	8 (27.5%)	1 (5%)
Mild	8 (27.5%)	14 (66%)
Moderate	11 (38%)	5 (24%)
Severe	2 (7%)	1 (5%)
Comorbidities		
HTN	3 (10%)	1 (5%)
Anxiety	3 (10%)	1 (5%)
Diabetes	1 (3.5%)	1 (5%)
Asthma	0	1 (5%)

HTN, Hypertension

### Simple plex assay

Plasma concentration of the inflammasome signaling proteins (Caspase-1, ASC, IL-1β and IL-18) were measured in 29 patients diagnosed with COVID-19, 21 patients who recovered from COVID-19 and 47 healthy controls who were never infected with COVID-19 as described in (16) using the Ella System (Protein Simple). Assays were run with the Runner Software (version 3.7.2.0) and automatically analyzed with the Simple Plex Explorer Software (version 3.7.2.0) (19).

### Statistical & biomarker analysis

Data were analyzed utilizing Prism 9.0 software (GraphPad). Descriptive statistics were run, and normality was tested by the Shapiro Wilk-Test or the D’Agostino & Pearson Test. Non-parametric data were analyzed correcting for multiple comparisons by controlling the False Discovery Rate (FDR) with the two-stage step-up method of Benjamini, Krieger and Yekutieli. P- and q-values of significance were set to p< 0.05.

Receiver operating characteristics (ROC) were calculated to obtain the area under the curve (AUC) and to determine cut-off points, specificity, sensitivity and the likelihood ratio as described in (15). Positive and negative predictive values were calculated along with accuracy. ROC were calculated for each analyte for healthy controls vs. COVID-19 positive patients.

Comparison of ROC curves between inflammatory biomarkers was carried out as described (20) using the following formula to obtain a critical ratio Z:


$$z=\frac{\left(A_{1}-A_{2}\right)}{\sqrt{SE_{1}^{\;2}+SE_{2}^{\;2}-2rSE_{1}SE_{2}}}$$

The p-value was determined using the following formula using Microsoft Excel (version 16.61.1):


=2∗(1−NORMSDIST(z))

A Spearman r correlation was used to obtain r in order to calculate the z-score to allow for comparison of ROC curves between analytes obtained from the same samples for different biomarkers.

## Results

### Inflammasome proteins are elevated in the blood of patients with COVID-19

**Figure 1:**
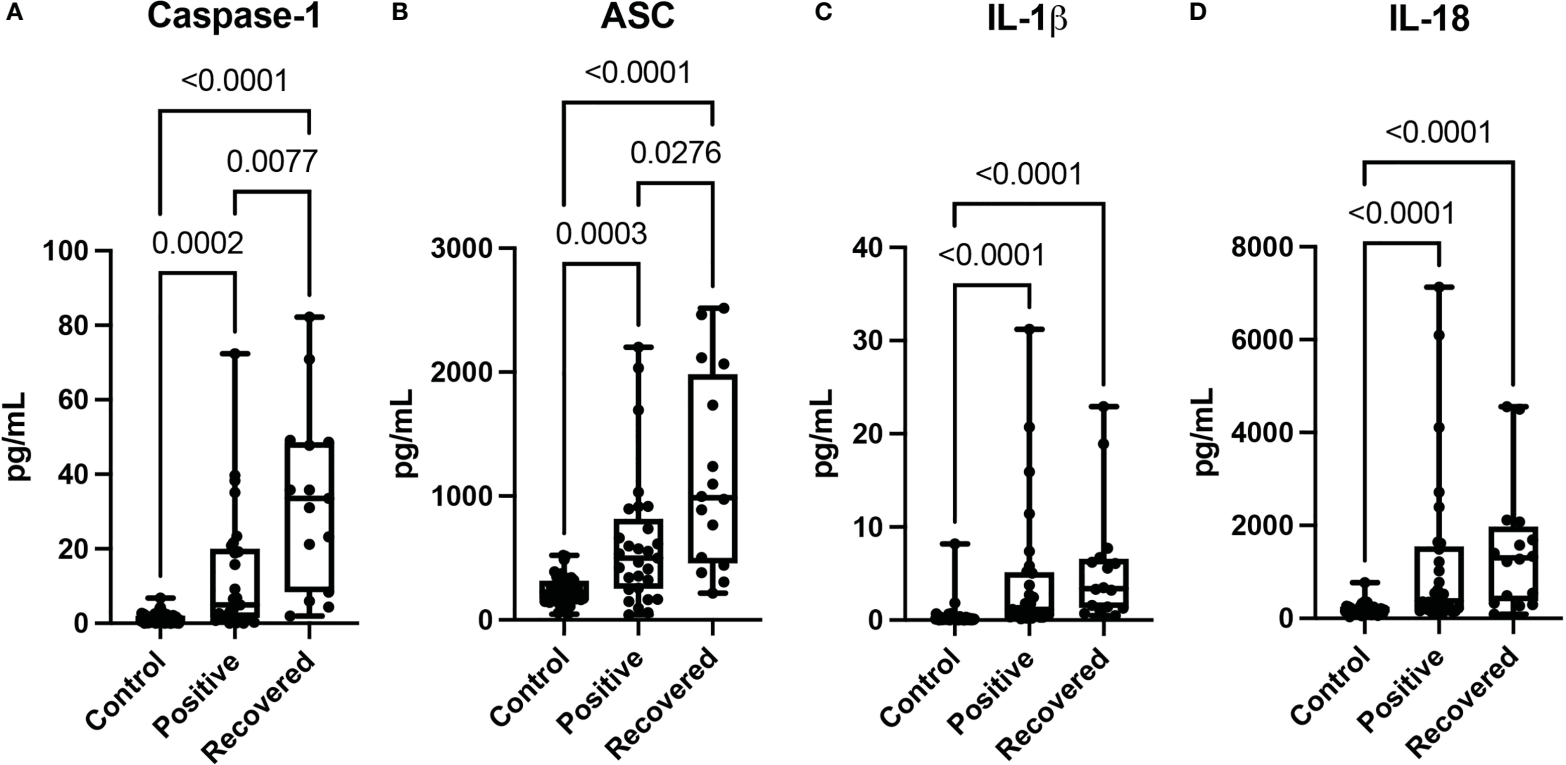
Inflammasome proteins after COVID-19 infection. Simple Plex Assay of blood plasma from patients with an active COVID-19 infection (Positive), patients recovered from COVID-19 (Recovered) and healthy controls who never had a COVID-19 infection (Control). Box and whiskers plots show the whiskers with the minimum, maximum and all data points for each inflammatory protein of interest with respective p values listed above. **(A)** Caspase-1: N: Control: 47, Positive: 29, Recovered: 15; **(B)** ASC: N: Control: 47, Positive: 29, Recovered: 16; **(C)** IL-1β: N: Control: 47, Positive: 29, Recovered: 16; and **(D)** IL-18: N: Control: 47, Positive: 29, Recovered: 16.

The inflammasome participates in the inflammatory response associated with coronavirus infections (2). We analyzed the protein levels of the inflammasome signaling proteins caspase-1 (**Figure 1A**), ASC (**Figure 1B**), IL-1β (**Figure 1C**) and IL-18 (**Figure 1D**) in patients with COVID-19 and patients who had recently recovered from a COVID-19 infection and compared them to the levels in plasma samples from healthy donors, who never had COVID-19. When compared to non-infected controls, protein levels of caspase-1 (**Figure 1A**), ASC (**Figure 1B**), IL-1β (**Figure 1C**) and IL-18 (**Figure 1D**) were higher in patients with active (Positive) COVID-19 (median day of blood collection: 11 days) and in patients who recently recovered from COVID-19 (median day of blood collection: 61.5 days). Moreover, the protein levels of caspase-1 (**Figure 1A**), and ASC (**Figure 1B**) were even higher in recovered patients than in individuals with an active COVID-19 infection (Positive). However, there was no statistical difference in the levels of IL-1β (**Figure 1C**) and IL-18 (**Figure 1D**) in recovered individuals when compared to those individuals who had an active infection, suggesting that capase-1 and ASC play an important role in more chronic inflammatory responses in patients who recovered from COVID-19 infection.

### Inflammatory biomarkers of COVID-19

**Figure 2:**
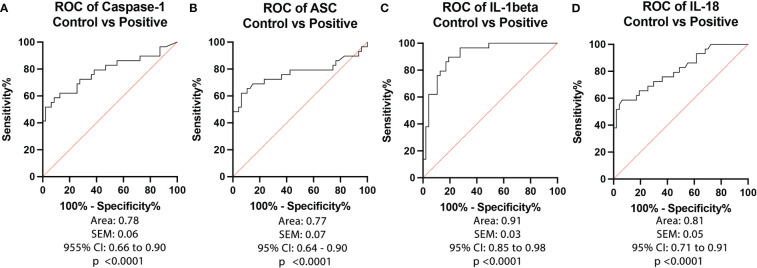
ROC of Inflammasome Biomarkers of active COVID-19 infection. ROC and AUC were calculated for each inflammasome signaling protein in the plasma of patients with an active COVID-19 infection (Positive) and healthy controls who never had a COVID-19 infection (Control). **(A)** Caspase-1: N: Control: 47, Positive: 29; **(B)** ASC: N: Control: 47, Positive: 29; **(C)** IL-1β: N: Control: 47, Positive: 29; and **(D)** IL-18: N: Control: 47, Positive: 29.

To determine the biomarker reliability of the inflammasome signaling proteins caspase-1 (**Figure 2A**), ASC (**Figure 2B**), IL-1β (**Figure 2C**) and IL-18 (**Figure 2D**), we plotted ROC curves for each protein using data from healthy subjects and from patients that tested positive for COVID-19 and presented an active infection (**Figure 2**). Accordingly, the best biomarker was IL-1β with an AUC of 0.91 (**Figure 2C**) followed by IL-18 with an AUC of 0.81 (**Figure 2D**), Caspase-1 with an AUC of 0.78 (**Figure 2B**), and ASC with an AUC of 0.77 (**Figure 2A**). IL-1β had the highest sensitivity of 90% with a 81% specificity at a cut-off point of 0.30 pg/mL, and 84% accuracy (**Table 2**), whereas caspase-1 had a sensitivity of 79% with an 62% specificity at a cut-off point of 1.4 pg/mL (**Table 2**), ASC presented 76% sensitivity and 64% specificity at a cut-off point of 255 pg/mL, and IL-18 with a cut-off point of 190 pg/mL, a 76% sensitivity and 64% specificity. Taken together, these data indicate that IL-1β, caspase-1, ASC and IL-18 are reliable biomarkers of COVID-19.

**Table 2:** Cut-off point in plasma of COVID-19 positive patients.

Biomarker	Cut-off point (pg/mL)	Sensitivity (%)	Specificity (%)	LR	PPV (%)	NPV (%)	Accuracy (%)
**Caspase-1**	> 1.39	79	62	2.071	56	83	68
**ASC**	> 255	76	64	2.097	56	81	68
**IL-1β**	> 0.2960	90	81	4.682	74	93	84
**IL-18**	> 190.0	76	64	2.097	66	74	70

**Figure 3:**
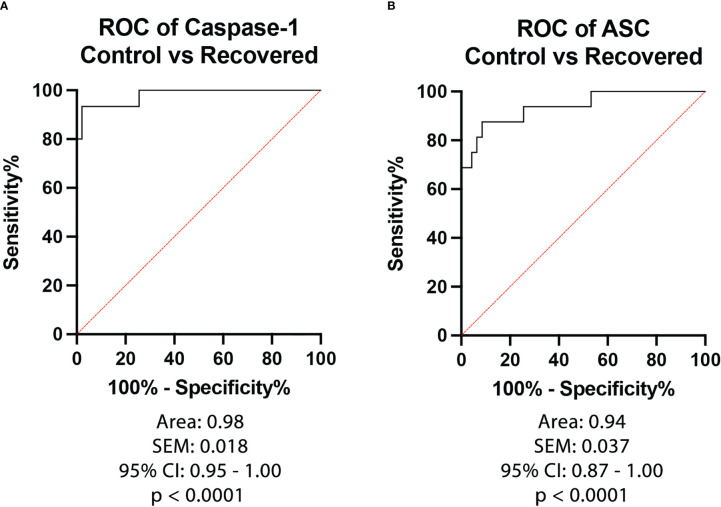
ROC of Inflammasome Biomarkers after recovery from COVID-19. ROC and AUC were calculated for each inflammasome signaling protein in the plasma of patients who recovered from an active COVID-19 infection (Positive) and healthy controls who never had a COVID-19 infection (Control). **(A)** Caspase-1: N: Control: 47, Recovered: 15; **(B)** ASC: N: Control: 47, Recovered: 16.

To then determine the biomarker reliability of the inflammasome signaling proteins that were significantly elevated after recovery from active infection, ROC curves were plotted for caspase-1 (**Figure 3A**) and ASC (**Figure 3B**) using data from healthy subjects and from patients that recovered from COVID-19 (**Figure 3**). Accordingly with an AUC of 0.98, a sensitivity of 93% and a specificity of 98% for caspase-1 and an AUC of 0.94, a sensitivity of 88% and a specificity of 91% for ASC, our findings indicate that caspase-1 and ASC are very good biomarkers of the inflammatory response taking place after recovery from COVID-19 infection (**Table 3**).

**Table 3:** Cut-off point in plasma of COVID-19 recovered patients.

Biomarker	Cut-off point (pg/mL)	Sensitivity (%)	Specificity (%)	LR	PPV (%)	NPV (%)	Accuracy (%)
**Caspase-1**	> 4.34	93	98	43.87	93	98	97
**ASC**	> 376.5	88	91	10.28	86	92	90

### ROC curve comparison

**Figure 4:**
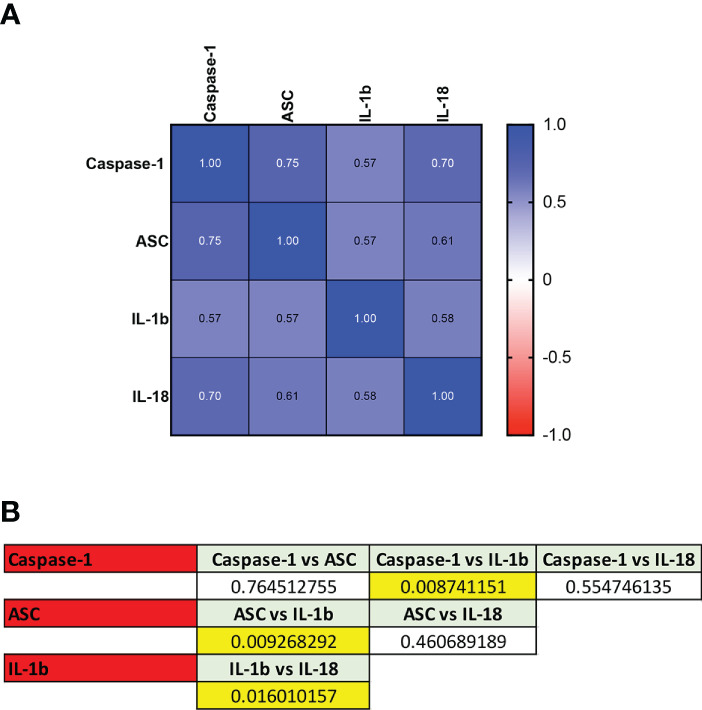
ROC Comparison among Inflammasome Biomarkers in COVID-19. **(A)** Correlation matrix using a Spearman correlation among biomarkers. **(B)** p-values of significance for the comparison among the biomarkers caspase-1, ASC, IL-1β and IL-18.

To compare the ROC curves for the different biomarkers identified, a Spearman correlation was carried out and a correlation matrix was plotted for the control vs positive cohorts (**Figure 4**). The highest correlation was found between caspase-1 and ASC (r= 0.75, p=1.12e-14) followed by caspase-1 and IL-18 (r= 0.70, p=1.81e-12), ASC and IL-18 (r= 0.61, p=4.25e-9), IL-1β and IL-18 (r= 0.58, p= 4.89e-8), ASC and IL-1β (r= 0.57, p=1.05e-7), and caspase-1 and IL-1β (r= 0.57, p=6.59e-8).

After determining the coefficient of correlation, the ROC curves were compared using the following formula:


$$z=\frac{\left(A_{1}-A_{2}\right)}{\sqrt{SE_{1}^{\;2}+SE_{2}^{\;2}-2rSE_{1}SE_{2}}}$$

Accordingly, ROC curve comparison analyses showed that the ROC curves differed between caspase-1 and IL-1β (p = 0.009), ASC and IL-1β (p = 0.009), and IL-1β and IL-18 (p= 0.02), Together, these findings indicate that when comparing biomarkers based on the ROC curves, IL-1β and IL-18 are different biomarkers with the potential to provide distinct information regarding the inflammatory response in COVID-19 infections, as well as either ASC or caspase-1 when compared to IL-1β. However, ASC and caspase-1, are similar biomarkers of the inflammatory response during active COVID-19 infection.

When comparing the ROC curve between caspase-1 and ASC for healthy controls vs recovered controls, the correlation was found to be 0.81 (p< 0.0001), and the curves were not different between each other, indicating that ASC and caspase-1 are similar biomarkers of the inflammatory response after active COVID-19 infection.

## Discussion

COVID-19 quickly became a pandemic with unprecedented consequences to public health and medicine (21). Despite the importance of COVID-19 to public health and safety, we do not fully understand how the inflammatory response contributes to the acute and chronic effects following SARS-CoV-2 infection.

Heightened inflammation is a major contributor to many of the deleterious and lethal effects associated with COVID-19. In addition, COVID-19 presents with potentially long-term sequelae. In this study, we have analyzed the protein profile of inflammasome signaling proteins in the plasma of patients with an active SARS-CoV-2 infection as well as patients who had recently recovered from the infection.

COVID-19 may result in a cytokine storm characterized by an excessive dysregulated inflammatory response (22). Ideally, the immune response acts to limit the spread of infection by eliminating virus-infected cells following sensing of pathogen-associated molecular patterns (PAMPs) by pattern recognition receptors (PRRs) that activate the innate immune response. An important pathway of the innate immune response is the NLRP3 inflammasome, that in the context of coronavirus infections, has been shown to be activated, in part, by a mechanism involving the envelop (E) protein of coronaviruses and the ion channel ORF3a (23). In this study, we found that the protein levels of the inflammasome signaling protein caspase-1, ASC, IL-1β and IL-18 were elevated during COVID-19 infection and remained elevated even after the active infection was over. The median time-point of blood collection in recovered patients was 61.5 days, and the range was from 28 days to 217 days, suggesting a chronic inflammatory response that is heightened for at least approximately 2 months after infection onset. Interestingly, the levels of caspase-1 (median = 33.50 pg/mL) and the adaptor protein ASC (median = 985 pg/mL) were even higher in the recovered patients than in patients with an active infection (4.92 and 498 pg/mL, respectively), whereas the levels of caspase-1 (0.89 pg/mL) and ASC (226 pg/mL) in healthy controls were lower than in the two other groups. Interestingly, the plasma levels of ASC in COVID-19 patients with active infection were lower than previously evaluated in patients with traumatic brain injury (619 pg/mL) (10), but these levels were higher in recovered patients than in traumatic brain injury patients. However, the levels of ASC in patients with active infection were similar to those found in patients with depression (452 pg/mL) (24) and Alzheimer’s Disease (452 pg/mL) (13), but less than patients with multiple sclerosis (538 pg/mL) (11), non-alcoholic steatohepatitis (644 pg/mL) (14), lupus nephritis (650 pg/mL) (16), psoriasis (674 pg/mL) (19) and diabetic kidney disease (918 pg/mL) (16).

Taken together, these data suggest that even after recovery, caspase-1 and ASC play a role in the chronic inflammatory immune response that might further exacerbate the long-term effects of COVID-19. However, future studies are needed to understand how these inflammasome proteins contribute to long-term sequelae after COVID-19 (2) since elevated levels of inflammasome proteins have been associated with a variety of diseases affecting the brain (7, 10–12, 25), the eyes (15) and the skin (19) as well as metabolic (14) and renal diseases (16), many of which have been associated with COVID-19 (2). Furthermore, the IL-1β levels in this study are consistent with a previous study that showed IL-1β values in the serum of hospitalized COVID-19 patients at approximately 0.7 pg/mL (26), whereas here we found values in plasma with a median of 0.132 pg/mL in healthy controls, 1.15 pg/mL for the Positive cohort and 3.395 pg/mL for the Recovered cohort, which although not statistically significant between the positive and recovered groups, it suggests a continuing increase in the levels of IL-1β over time that mimics the increase seen in ASC and caspase-1 protein levels in recovered individuals, which also mimics the trend detected for IL-18 (Control: 161.2 pg/mL, Positive: 379 pg/mL, Recovered: 1,306 pg/mL). Moreover, it is possible that the difference in IL-1β levels is due to the type of fluid used in the different studies. Accordingly, in this study we used plasma and other studies have used serum, suggesting that considering the difficulty of measuring IL-1β in body fluids, plasma collected in EDTA tubes might be a better choice for IL-1β analysis than measuring IL-1β in serum.

Elevation of IL-1β, TNF and IL-6 may contribute to the cytokine storm in patients with severe COVID-19 (27). Although a variety of cells play a role in the release of inflammasome proteins and inflammatory cytokines, endothelial cells are main producers of these proteins. Endothelial cells are found in the inner lining of blood vessels and play a significant role in vascular function, including blood clotting (28), which has been described in patients with COVID-19.

Furthermore, the comorbidities associated with severe COVID-19 such as hypertension, diabetes, cardiovascular disease and obesity highlight the importance of the vascular system and endothelial cell dysfunction to COVID-19 pathology. The heightened inflammatory response associated with COVID-19 shifts endothelial cells into a pro-inflammatory phenotype with high permeability and pro-thrombic characteristics (29, 30). Here, we have run analyses for the association between inflammasome biomarkers and disease severity as well as sex differences, and we did not find an association between the different variables. These results are consistent with a previous study that analyzed IL-1β in the serum of 71 COVID-19 patients showing that there was no statistical significant difference in IL-1β levels between COVID-19 patients who survived and those who died, suggesting that IL-1β levels in blood do not correlate with disease severity (31). However, a previous report has shown that caspase-1 and IL-18 in serum correlate with COVID-19 severity (32). Moreover, we did not find a relationship between comorbidities and the protein levels of the biomarkers analyzed. Future studies, powered for different disease severities and for patients presenting different comorbidities are needed to determine whether inflammasome signaling proteins are associated with disease severity and whether comorbidities affect inflammasome protein levels associated with COVID-19.

Our study was designed to evaluate the reliability of biomarkers that may be used to provide clinical parameters to measure the acute and chronic inflammatory consequences of COVID-19. Identification of biomarkers that provide information about the inflammatory status of the disease are important milestones towards the identification of theragnostic biomarkers that can aid clinicians in the monitoring of response to treatment as well as in the identification of future therapeutic approaches that can be used in the care of COVID-19 patients. Our results show that the inflammasome signaling proteins IL-1β with an AUC of 0.91, and IL-18 with an AUC of 0.81 are the two most reliable biomarkers of the inflammatory response in COVID-19 among the inflammasome proteins analyzed in this study. However, with AUC values above 0.7 for caspase-1 and ASC, our findings indicate that these proteins are also reliable biomarkers of the inflammatory response in COVID-19. Furthermore, given that caspase-1 and ASC protein levels keep on increasing after recovery, our findings suggest that these two inflammasome proteins are even better biomarkers of the inflammatory response after recovery. Moreover, ROC curve comparison between the different analytes indicates that when comparing biomarkers based on the ROC curves, IL-1β and IL-18 have the potential to provide distinct information regarding the inflammatory response in active COVID-19 infections, as well as either ASC or caspase-1 when compared to IL-1β. However, ASC and caspase-1, seem to be similar biomarkers.

According to Samprathi and Jayashree, the potential identification of biomarkers of COVID-19 would provide many benefits for the care of these patients such as the potential to confirm early disease, confirm and classify disease severity, frame hospital/intensive care unit admission/discharge criteria, identify high-risk groups, rationalize therapies and assess response to treatment as well as predict disease outcome (3).

To date, biomarkers that have been studied for COVID-19 include hematologic parameters such as anemia (33), platelets (34), ferritin:transferrin ratio (33), peripheral blood leukocyte and lymphocyte counts (35), absolute number of neutrophils (36), monocytes, basophils, eosinophils (37) and eosinophil-derived neurotoxin (EDN-1) levels (38). In regard to inflammatory proteins and their role as biomarkers of COVID-19, protein levels of C-reactive protein (CRP) has been shown to be elevated in approximately 60% of COVID-19 patients with a cut-off point greater than 10 mg/L. However, patients who progressed to severe disease presented a blood CRP with a cut-off point greater than 26 mg/mL (39). Moreover, inflammatory cytokines that have been shown to be elevated in patients with COVID-19 include IL-6, L-1β, IL-2, IL-8, IL-17, G-CSF, GMCSF, IP-10, MCP-1, CCL3 and TNFα, with IL-6 being considered a significant predictor of disease severity and mortality (3, 40).

In this study, although we did not analyze what variants of COVID-19 the subjects used were infected with, we have analyzed samples from patients who were infected between July 20^th^, 2020, and November 8^th^, 2020. Thus, we expect that these patients were most likely infected with the original form of the virus that started to spread in December 2019, and prior to the B.1327 and B.1429 variants that were described in July, 2020. Importantly, the evidence suggests that the original strain responsible for COVID-19 caused a significantly elevated inflammatory response, in part due to the lack of vaccination at that point. However, other subsequent variants, such as the delta variant, have also been described to manifest with a severe inflammatory response (41). Thus, future studies need to examine the inflammatory profile from the blood of patients infected with different variants during active infection and after recovery in order to better understand how inflammation contributes to the acute phase and shortly after recovery from the active infection due to infection with different strains of the virus. Furthermore, it would be important to determine the levels of these proteins in more severe cases to more moderate cases during active infection. However, here we did not detect any differences between the different severities of infection for any of the analytes studied (data not shown). Moreover, another limitation in this study is that we did not analyze the protein levels of these analytes in the same patients across the different groups tested.

Lastly, it is critical to better understand the long-term consequence in the inflammatory response in the more chronic stages after recovery, given the prevalence of post-COVID-19 syndrome (PCS), also known as long COVID (42). Since the symptoms associated with PCS include neurological, metabolic and cardiac problems (43), it is important to identify biomarkers of COVID-19 and PCS in order to develop better therapeutic strategies to treat the large patient population that in the future may develop diseases as a result of having recovered from severe COVID-19 (44). Here, we did not detect a correlation between the protein levels of these analytes and the days after infection. However, future studies focusing on patients with long-COVID will be needed to determine whether there is a correlation between the levels of inflammasome signaling proteins with recovery time.

Taken together, our study has identified inflammasome proteins as reliable biomarkers of the inflammatory response in COVID-19 pathology. However, studies with a larger cohort of patients and patients with different disease severity and treatments are needed to further evaluate whether biomarkers of the inflammatory response are also reliable prognostic (disease severity) and theragnostic (response to treatment) biomarkers of COVID-19 pathology.

Furthermore, this study also indicates that these biomarkers remain elevated for at least approximately two months following infection. Thus, highlighting the need for chronic studies into the long-term effects of COVID-19 and PCS since due to the pandemic nature of this infection, the number of affected individuals who may develop long term complications due to a sustained chronic inflammatory response is significant. Therefore, therapeutics targeting these pathological processes may show efficacy in reducing the chronic complications of this disease.

## Data availability statement

The raw data supporting the conclusions of this article will be made available by the authors, without undue reservation.

## Ethics statement

The studies involving human participants were reviewed and approved by SERATRIALS, LLC - IRB number: 20200828. The patients/participants provided their written informed consent to participate in this study.

## Author contributions

RH and JR performed the research. RWK and JPdRV designed the research study. All authors contributed to the article and approved the submitted version.
